# A decision tree model suggests a strong interaction effect between tumor size and a close surgical margin on the prognosis of limb salvage surgery in high-grade osteosarcoma

**DOI:** 10.3389/fsurg.2026.1801218

**Published:** 2026-04-24

**Authors:** Yingying Shi, Lan Wei, Qiyuan Bao, Junxiang Wen, Zhusheng Zhang, Zhuochao Liu, Qi Liu, Jie Chen, Xiaoqing Yang

**Affiliations:** 1 Department of Operating Room, Ruijin Hospital Affiliated to Shanghai Jiao Tong University School of Medicine, Shanghai, China; 2 Department of Nursing, Ruijin Hospital Affiliated to Shanghai Jiao Tong University School of Medicine, Shanghai, China; 3 Department of Orthopedics, Shanghai Institute of Orthopedics and Traumatology, Shanghai Ruijin Hospital, Shanghai Jiaotong University School of Medicine, Shanghai, China; 4 Department of Thoracic Surgery, Shanghai Ruijin Hospital, Shanghai Jiaotong University School of Medicine, Shanghai, China

**Keywords:** decision tree, limb salvage surgery, osteosarcoma, peri-neurovascular margin, surgical margin

## Abstract

**Purpose:**

The effect of a close surgical margin (CSM) due to the preservation of vital structures in limb salvage surgery (LSS) for high-grade osteosarcoma (OS) is still controversial. This article aimed to study the prognosis of LSS with a close peri-neurovascular margin (PNM).

**Methods:**

We retrospectively reviewed 196 cases that underwent LSS from January 2010 to December 2015 at our institution, of which 88 involved high-grade extremity osteosarcoma with a potential risk of an “inadequate” wide margin (<15 mm) according to the Enneking classification system. Data on surgical and tumor-related factors were collected together with the width of the PNM. Survival analysis and machine learning (ML) algorithms with cross-validation were used to construct the prognostic model for oncological outcomes after LSS.

**Results:**

PNM was associated with a higher local recurrence (LR) rate, while greater tumor size was a risk factor for metastasis and overall survival in the multivariate Cox regression model. Using ML algorithms, a decision tree (DT) model was constructed, indicating that a closer PNM was significantly correlated with higher LR-free survival only when the tumor size was less, but not greater, than 31.6% of the limb (with a significant interaction effect). Interestingly, a conventional survival analysis and receiver operating characteristic plot confirmed the robust interaction effect of PNM and tumor size on not only LR, but also metastasis and overall survival, with an even larger effect size than that of each factor alone.

**Conclusion:**

The prognostic effect of CSM in high-grade osteosarcoma is dependent on the tumor size. We found that a close PNM (< 2.4 mm) in LSS due to the preservation of critical structures is a risk factor for LR and survival in patients with small-sized osteosarcomas. For a large-sized tumor, the prognosis is unsatisfactory regardless of the state of the CSM. Further studies are needed to explore the mechanisms underlying such heterogeneity in LSS.

## 1 Introduction

Limb salvage surgery (LSS) is the preferred modality for the surgical management of osteosarcoma (OS) (1). Complete resection with an appropriate surgical margin is a prerequisite for the success of local control of the disease and improved survival (2–4) and is perhaps the factor that could be the most affected by the surgeon’s skill (5).

However, given the broad consensus on the superiority of wide vs. marginal margins for OS, current opinions on the question of “how wide” are highly controversial (6, 7). Traditional opinion recommends a >1–2 cm healthy margin as the so-called “adequate” wide margin for *en bloc* resection of extremity bone and soft tissue sarcoma, while a wide margin less than that is considered “inadequate” (8, 9) and not recommended (10–12). However, the close proximity of the tumor to vessels/nerves usually seen in OS makes this concept challenging for treating surgeons, and prognosis assessment of different close surgical margins (CSMs) is needed (6, 13). In this regard, recent studies have investigated CSMs from 5 mm (14) to 1 mm (2, 3), and even super-close but pathologically negative margins (2, 14). These reports had contradictory findings, as no difference in oncological results was found for various “inadequately” wide surgical resections.

One possible reason for this may be the complex heterogeneity of OS (9, 15). The patient cohort may vary from study to study according to their specific aims. A heterogeneous population [e.g., amputation vs. limb salvage, peri-neurovascular margin (PNM) vs. soft tissue margin, definition of a close margin as 5 mm vs. 1 mm, and so on] may lead to high heterogeneity in the clinicopathological features of the sample. Additionally, the interactions among these clinicopathological factors could theoretically exist yet be underestimated when using conventional statistics, which assume linear relationships between the independent and dependent variables (16, 17).

To study the effects of surgical margins and other prognostic factors specific to CSM cases, we retrospectively reviewed 196 cases of high-grade extremity OS that underwent LSS in our institution and included wide resection cases with peri-neurovascular margins <15 mm. Candidate risk factors were then analyzed in the final cohort of 88 cases using survival analysis and machine learning (ML) models for the oncological outcome after surgery.

## 2 Materials and methods

### 2.1 Patient selection and clinical data

With the approval of the Institutional Research Review Board of Ruijin Hospital, Shanghai Jiaotong University School of Medicine, we retrospectively reviewed a total of 196 osteosarcoma patients in our database who underwent LSS and intraoperative neurovascular bundle dissection, excluding vascular resection and graft reconstruction, between January 2010 and December 2015. (1) Based on our criteria, we reviewed all pre-surgical imaging data and excluded 65 cases (33.2%) for having incomplete imaging data or imaging data showing a PNM greater than 15 mm. (2) Pathological records indicated 16 (8.2%) non-high-grade cases, of which four were intermediate-grade chondroblastic osteosarcomas, four were low-grade central OS, five were periosteal OS, one was secondary low-grade OS, and two were undetermined low-grade OS. (3) In three cases (1.5%), the postoperative pathology was inconclusive but was finally determined to be clear cell sarcoma (one case), Ewing sarcoma (one case), and an aneurysmal bone cyst (one case). (4) In seven (3.6%) patients, the malignancy involved the acetabular or pelvic regions. (5) Moreover, 10 cases were excluded due to having an intraoperative pathology-confirmed positive margin, or a marginal margin .(6) Subjects were also excluded if they received LSS outside our department (seven cases, 5.1%). Our final cohort comprised 88 patients with high-grade extremity osteosarcomas who were undergoing LSS with CSM, including those with Enneking stage IIB (localized) or III with manageable primary metastasis, for whom LSS was also performed with a curative purpose. The latter were included because this subset may have a similar prognosis if all known metastatic deposits are removed together with LSS (9). All patients underwent a “standardized” preoperative and postoperative chemotherapy regimen including doxorubicin, cisplatin, methotrexate, and ifosfamide according to the IOR/OS-N4 protocol (18, 19) based on the individual's body surface area. The collected demographic information includes age, gender, delay in treatment (from the onset of symptoms to the time of treatment), tumor location, laterality, concurrent metastasis at presentation, pathological fracture, and biopsy performed at another center. Informed consent had been previously obtained from all included subjects.

### 2.2 Tumor morphological data

Before surgery, MRI data were collected using a 1.5 T clinical scanner (SignaHDx, GE Medical Systems, Milwaukee, Wisconsin, USA) with a longitudinal T1-weighted spin echo (SE) [repetition time (TR)\echo time (TE) 860/12 ms] to determine the intramedullary axial length of the tumor. With a field of view of 140–160 mm, a slice thickness of 4.0 mm, and a voxel size of 0.6 mm × 0.6 mm × 4.0 mm, traverse T2-weighted fast SE (TR/TE 4500/132 ms) and multiple train-echo short-tau inversion recovery (TR/TE 3500/18) were used to measure the following tumor morphology: (a) tumor cross-sectional area, determined by manual segmentation of the tumor on the traverse slice across the largest portion of the tumor; (b) percentage of tumor cross-sectional area (PTA), defined as the tumor cross-sectional area normalized by the cross-sectional area of the limb at the same slice; (c) height, width, and depth, which were measured, and tumor volume, which was calculated based on the formula “V = π/6 × height×width×depth” (20). In addition, the reactive zone was visualized and the thickest part was measured, and the PNM was measured as the shortest distance from the outer border of the tumor pseudocapsule to the sheath of the neurovascular bundle to be preserved. All of these metrics were repeated and averaged on three consecutive planes.

### 2.3 Limb salvage surgery

In cases with a CSM, the implementation of wide resection is most commonly compromised by the need to preserve major nerves/vessels, as has been previously reported (7, 21–23) and in our experience. Thus, our pre-surgical planning in these cases was mainly focused on the available margin next to the neurovascular bundle to be preserved, which was preoperatively identified by MRI and angiography. Intraoperatively, deliberate dissection of the peri-neurovascular fat layer was first conducted to guarantee a safe margin on this side. Subsequently, on the other side, a regular >2 cm margin of bone and soft tissue was then achieved. All the surgical procedures and preoperative workup were led by a single experienced surgeon (W.B.Z.). Wide margins were intended for the majority of the LSSs, except in a few cases where the normal tissue cuff between the tumor and the neurovascular bundle was equivocal, and the patient had a strong desire for limb preservation despite the risk of disease progression.

### 2.4 Survival analysis and machine learning algorithms

In the univariate analyses, local recurrence (LR)-free survival, metastasis-free survival (MFS), and overall survival were compared using the Kaplan–Meier method (the log-rank test) for the categorical variables and the univariate Cox proportional hazard model for the continuous variables. A multivariate analysis was then performed using the Cox proportional hazards model and backward selection. Time to event was defined as the time from the date of LSS to the date of the event. Descriptive statistics are presented as median (range). Factors with a *P*-value less than 0.05 were considered significant.

Subsequently, ML algorithms, namely, naïve Bayes (NB), decision tree (DT), support vector machine (SVM), and TreeBagger (TB), were used to construct a classification model, with recurrence or non-recurrence at a given time point serving as the dichotomous label. To prevent overfitting, a five-fold cross-validation method was applied for model comparison and feature selection. Briefly, we first included all pre-surgical factors in the training set and evaluated the classification accuracy of various classifiers to compare these algorithms at a gross level. Second, for each algorithm, we performed cross-validation-based sequential feature selection to choose a set of attributes with generalizability. Finally, we fed all the data into the final model to generate an accuracy and confusion matrix (see Supplementary Methods for details). IBM SPSS 20 and MATLAB software were used for the statistical analyses.

## 3 Results

### 3.1 Oncological results and prognostic factors following LSS

Of the 88 patients, a PNM of less than 6 mm was found in the majority (51 of 88) of the cases (Supplementary Figure 1A), with a median PNM of 4.7 mm (range: 0.4–14.9). After a median follow-up of 29.8 months (range: 12–129), the 2- and 5-year LR-free survival rates in the cohort were 76.0 ± 5.2% and 65.4 ± 6.7%, respectively. Moreover, 67.5 ± 5.5% and 0.56 ± 6.0% of the patients were metastasis-free at 2 and 5 years after their surgery. The overall survival at 2 and 5 years was found to be 82.8 ± 4.4% and 64.4 ± 6. 7%, respectively **(**Supplementary Figures 1B–C).

Descriptive statistics and univariate and multivariate analyses of local recurrence-free survival (LRFS), MFS, and overall survival for each variable are presented in Table 1. Specifically, a wider PNM (HR = 1.01, *p* < 0.01) and having a tumor located at the proximal femur (HR = 4.54, *p* = 0.04) or proximal humerus (HR = 5.54,*p* = 0.01） were associated with a higher LRFS rate, with PNM remaining significant in the multivariate Cox proportional hazard model. The univariate analysis of MFS indicated intramedullary axial length (HR = 1.21, *p* = 0.04), PTA (HR = 1.02, *p* = 0.04), and tumor diameter > 8 cm (HR = 2.13, *p* = 0.03) were associated factors, with PTA remaining significant in the multivariate analysis. Furthermore, a greater PTA (HR = 1.03, *p* = 0.03), Enneking staging (III vs. IIB) (HR = 3.93, *p* = 0.02), and a tumor diameter >8 cm (HR = 2.38, *p* = 0.04) were found to correlate with worse overall survival post-surgery in the univariate analysis, with PTA still significant (*p* < 0.05) and Enneking staging borderline significant (*p* = 0.06) in the multivariate model.

**Table 1:** Univariate and multivariate analyses of local recurrence, metastasis, and overall survival after LSS.

	Median (range)/ No. (percentage)	LRFS		MFS		Overall survival	
Factors		HR	*p*	HR	*p*	HR	*p*
Delay in treatment (months)	3.0 (0.1–15.0)	0.89	0.31	0.97	0.65	0.99	0.87
Age (years)	18.0 (10.0–74.0)	1.02	0.24	0.99	0.77	1.01	0.89
Axial length (cm)	5.4 (1.6–10.5)	1.04	0.73	1.21	**0** **.** **04**	1.23	0.08
Cross-sectional area (cm^2^)	41.3 (6.3–201.8)	1.01	0.66	1.01	0.99	1.00	0.94
Percentage of cross-sectional area (%)	28.0 (7.2–75.7)	1.03	0.09	1.02	0.04#	1.03	**0.03**#
Tumor volume (cm^3^)	191.1 (18.6–2071.6)	1.01	0.19	1.01	0.13	1.01	0.10
Peri-neurovascular margin (mm)	4.7 (0.4–14.9)	1.01	**0.01**#	0.96	0.32	0.94	0.24
Reactive zone (mm)	4.2 (0.0–37.1)	1.06	0.84	0.78	0.38		
Gender							
Male subjects	52 (59.1%)	Ref					
Female subjects	36 (40.9%)	0.63	0.33	1.12	0.75	1.15	0.76
Age							
≤18 years)	48 (54.5%)	Ref					
>18 years)	40 (45.5%)	0.77	0.56	0.65	0.24	0.61	0.26
Surgery site							
Tibia proximal	33 (37.5%)	Ref					
Femur distal	37 (45.3%)	2.77	0.09	1.14	0.74	0.63	0.34
Femur proximal	6 (6.8%)	4.54	**0** **.** **04**	0.72	0.67	0.36	0.33
Humerus proximal	8 (9.1%)	5.54	**0** **.** **01**	1.44	0.57	0.50	0.50
Fibula proximal	4 (4.5%)	1.76	0.61	0.00	0.98	0.00	0.98
Laterality							
Left	37 (38.9%)	Ref					
Right	58 (61.1%)	0.70	0.42	0.70	0.33	0.83	0.29
Enneking staging							
IIB	83 (94.3%)						
III	5 (5.6%)	1.08	0.94	2.73	0.09	3.93	0.02*
Pathological fracture							
No	82 (93.2%)						
Yes	6 (6.8%)	1.14	0.88	1.34	0.69	1.59	0.66
Biopsy procedure at another center							
No	66 (75%)						
Yes	22 (25%)	1.19	0.74	1.17	0.70	1.09	0.87
Tumor diameter							
≤8 cm	61 (69.3%)	Ref					
>8 cm	27 (30.7%)	1.01	0.98	2.13	**0** **.** **04**	2.38	**0** **.** **04**
Proximal extremity (HP, FP)	14 (15.9%)	Ref					
Distal extremity (FD, TP, FiP)	74 (84.1%)	2.70	**0** **.** **03**	1.04	0.94	0.55	0.42

Categorical variables were compared using the log-rank test, and continuous variables and hazard ratios were analyzed using the Cox proportional hazards model. Variables with a two-tailed *p*-value no greater than 0.1 in the univariate analysis were included in the multivariate analysis. The *p*-values for each variable in the univariate analysis are shown with significant factors (*p* < 0.05) in bold. Factors that remained significant (borderline) in the multivariate analysis are noted with a hashtag (#) for *p* < 0.05 and an asterisk (*) for *p* = 0.06, respectively. LRFS, local recurrence-free survival; MFS, metastasis-free survival; HP, humerus proximal; FP, femur proximal; FD, femur distal; TP, tibia proximal; FiP, fibula proximal; HR, hazard ratio; Ref, reference.

### 3.2 Using machine learning algorithms to model postoperative LR

**Figure 1:**
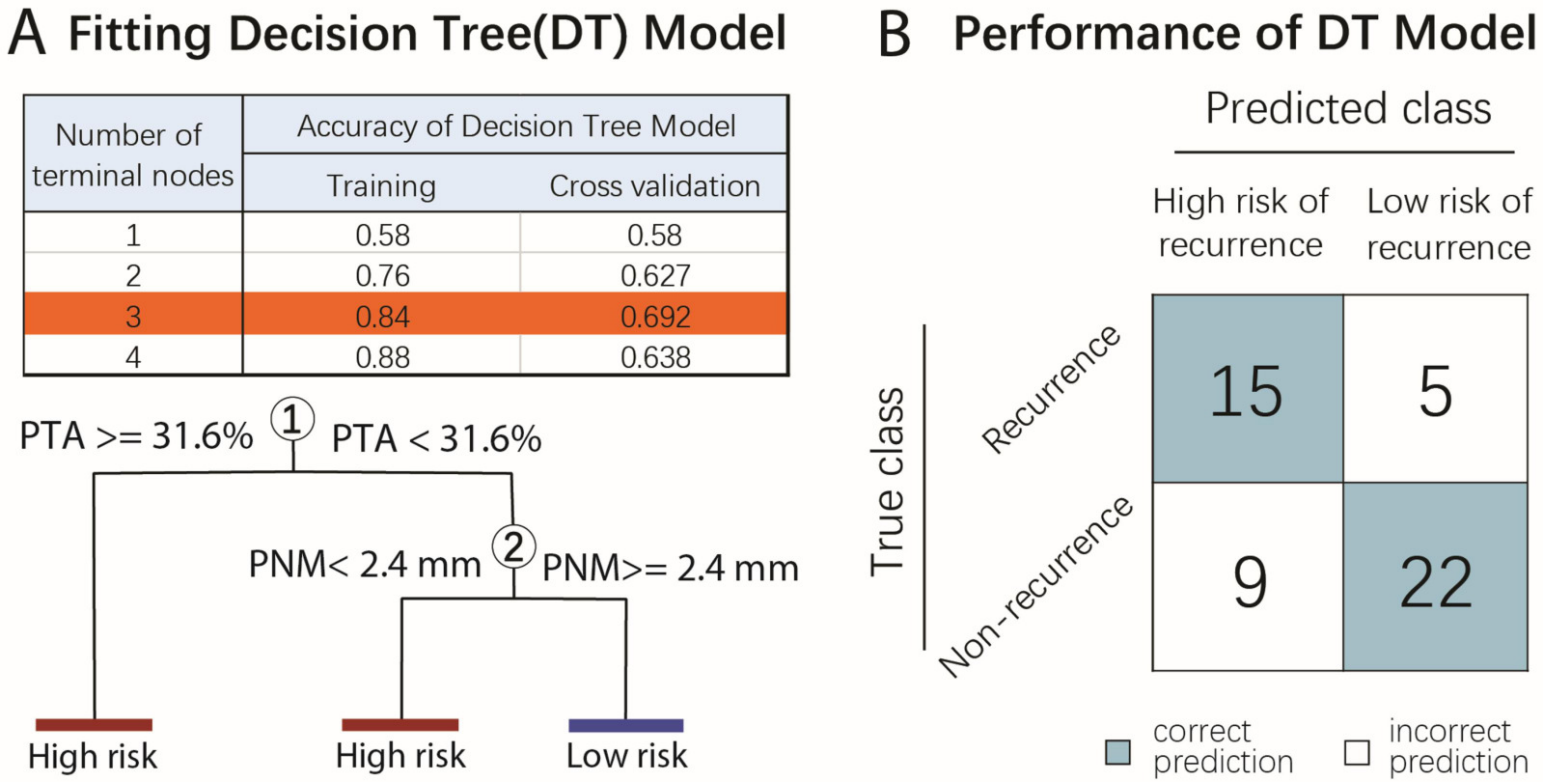
Prognostic model using the DT algorithm to predict tumor LR at 30 months after LSS. **(A)** The cross-validation method was used to fit the DT model. The concurrence of a wider PNM (≥2.4 mm) and a smaller tumor size (PTA < 31.6%) were predictive of a low risk of LR, while either a closer PNM (<2.4 mm) or a larger PTA (≥31.6%) were indicative of a high probability of LR. **(B)** The predictive power of the DT model is demonstrated by the confusion matrix. Correct predictions (blue) were achieved in 15 of the 20 recurrence cases and 22 of the 27 non-recurrence cases. PNM, peri-neurovascular margin; PTA, percentage of tumor cross-sectional area.

Since LR is reported to be significantly associated with surgical margins, its effect on survival remains controversial (6). We input our surgical and tumor-related parameters into a multivariate classification model with various ML algorithms and found that the prediction accuracy of the NB, DT, SVM, and TB algorithms was consistently around 70%, which was superior to the conventional Cox regression model (54.7%) (see Supplementary Table 1, Supplementary Figure 2). Given the similar predictive power of the different algorithms, we then chose the most simplistic and interpretable one, namely DT, as our final model, consistent with the commonly accepted recommendation (24–26). Using the cross-validation method, a DT model with three terminal nodes was found to have 70% accuracy in the cross-validation set (Figure 1A, Supplementary Figure 3).

In this model, concurrence of a wider PNM (≥2.4 mm) and smaller tumor size (PTA < 31.6%) were predictive of a low risk of LR, while either a closer PNM (<2.4 mm) or a greater PTA (≥31.6%) were indicative of a high risk of LR (Figure 1A). The high-risk group had an LR hazard ratio of 6.33 compared with the low-risk group (*p* < 0.001, log-rank test). At 30 months post-surgery, resubstituting all the datasets into this model correctly identified 15 of the 20 recurrence cases and 22 of the 27 non-recurrence cases (Figure 1B), with an overall accuracy of 72.5%.

### 3.3 The interaction effect between margin and tumor size

**Figure 2:**
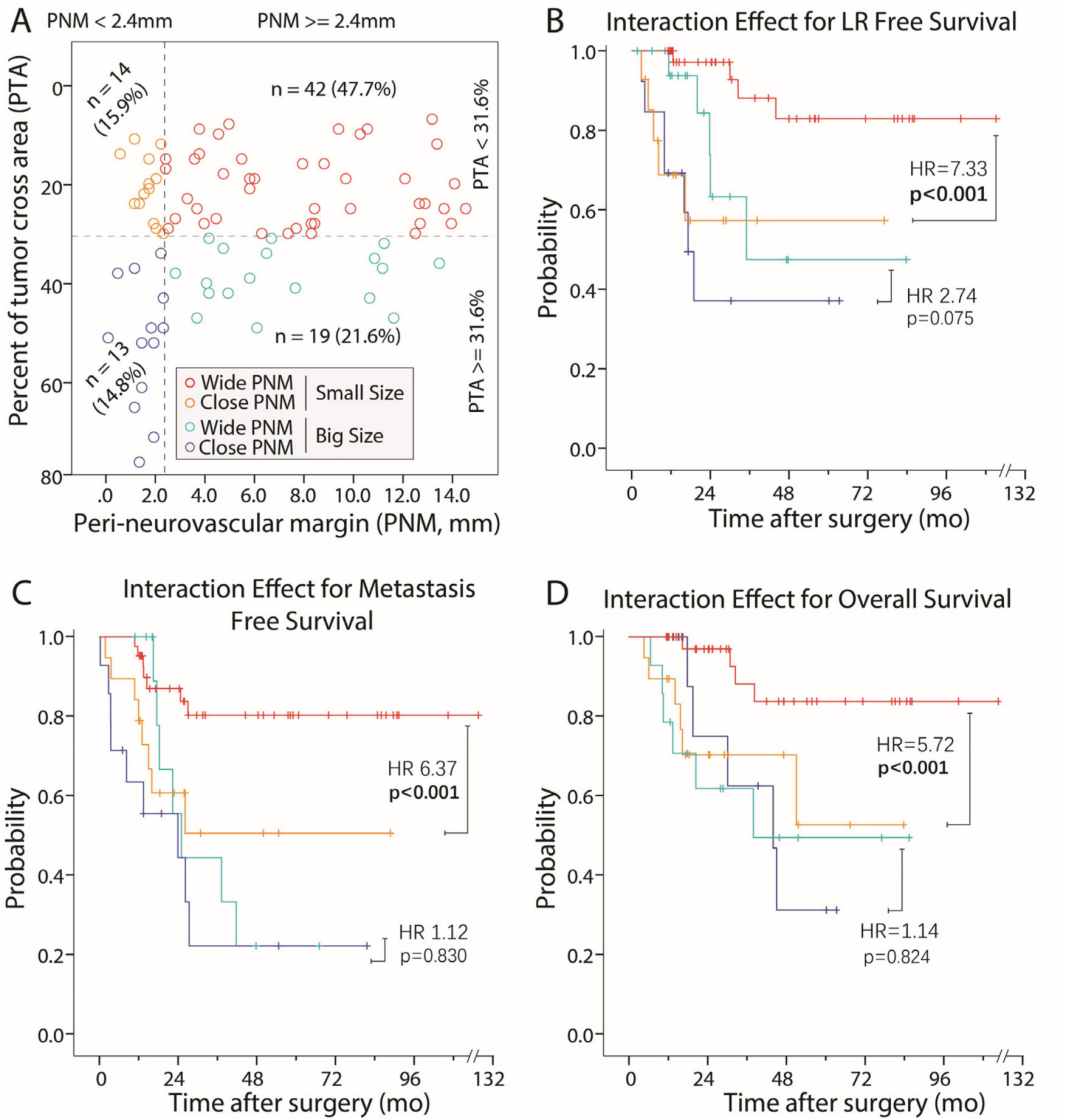
The interaction effect of the peri-neurovascular margin and tumor size (PTA). **(A)** Based on our DT model, all the patients were classified into one of four categories according to their PNM (whether ≥2.4 mm) and PTA (whether <31.6%). The number (proportion) of cases in each category is shown close to each scatterplot. **(B–D)** When the tumor was small (PTA < 31.6%), a significant difference between a close vs. wide PNM was found for LR (HR = 7.33, *p* < 0.001), metastasis (HR = 6.37, *p* < 0.001), and overall survival (HR = 5.72, *p* < 0.001). However, this difference was less significant (HR ranging from 1.14 to 2.74) and insignificant (*p* > 0.05) when the tumor was large (PTA ≥ 31.6%). PNM, peri-neurovascular margin; PTA, percentage of tumor cross-sectional area.

To validate the potential interaction effect of PNM and PTA suggested by our DT model, we stratified all the cases into four classes according to our DT model (Figure 2A), and compared their LRFS, MFS, and overall survival using a pairwise Kaplan–Meier analysis with a Bonferroni-corrected *p*-value of 0.008 (0.05/6). Our results clearly indicated that when the tumor cross-sectional area was small (PTA < 31.6%), a closer PNM was found to be strongly correlated with higher rates of LR (HR = 7.33, *p* < 0.001), metastasis (HR = 6.37, *p* < 0.001), and overall survival (HR = 5.72, *p* < 0.001). However, this difference was not significant with a larger tumor cross-sectional area (PTA ≥ 31.6%) (log-rank test *p* > 0.008), indicating that the effect of peri-neurovascular margins is dependent on the PTA (Figures 2B–D).

**Figure 3:**
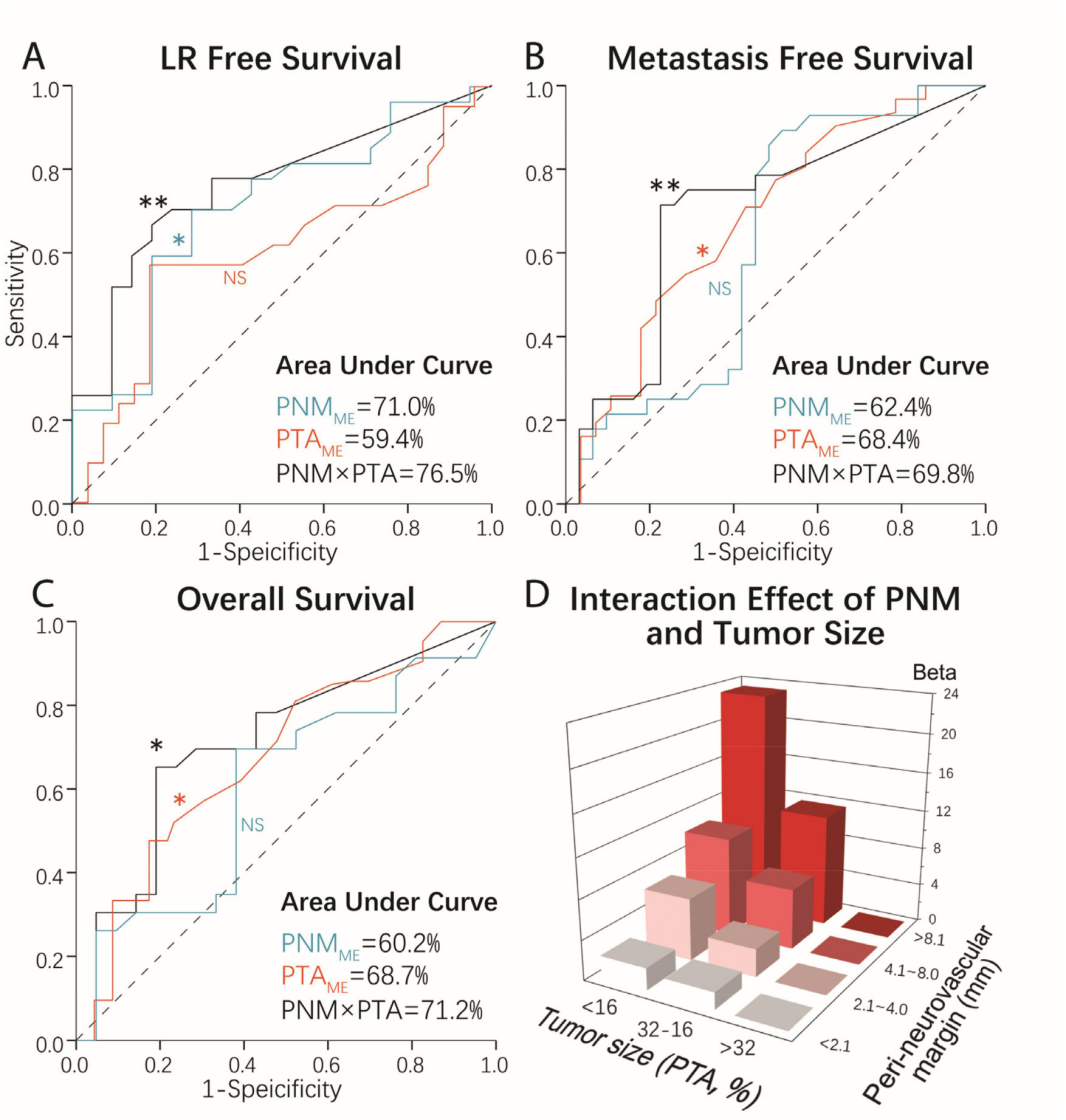
Comparing the effect size of the interaction effect and the main effects of margin and tumor size using a receiver operating characteristic plot. The AUC was compared between PNM, PTA, and their interaction term. PNM × PTA had an AUC of 76.5% for predicting LR **(A)**, an AUC of 69.8% for predicting metastasis **(B),** and an AUC of 71.2% for predicting overall survival **(C)** of the patients after LSS. A larger margin was associated with better oncological outcomes, given a smaller, but not a larger, tumor size. A visualization of the interaction effect of PNM and PTA is shown in **(D)**, with the horizontal axis signifying the Beta coefficient in the proportional hazard model, which equals −0.186 (HR = 0.830) for the LR rate, −0.102 (HR = 0.903) for the metastasis rate, and −0.122 (HR = 0.885) for mortality after surgery. PNM, peri-neurovascular margin; PTA, percentage of tumor cross-sectional area; ME, main effect, *p < 0.05, **p < 0.01, NS, not significant.

Furthermore, we re-validated this interaction effect using the conventional Cox proportional hazards model. We stratified tumor size based on PTA into big (>32%), medium (16%–32%), and small (<16%), and used this ordinal variable multiplied by PNM to form an interaction term. To avoid the multicollinearity problem, we inspected all the variables to ensure that none had correlation coefficients greater than 0.7 or a variance inflation factor greater than 5.0. Interestingly, adding PTA × PNM as an interaction term to the original multivariate model was significant (*p* < 0.05) in all the multivariate Cox proportional hazard models for LRFS, MFS, and overall survival (Table 2, Figure 3D). The relationship between tumor size and PTA was found to be non-linear, as demonstrated in Figure 3D, and the interaction had a more robust effect size compared with the main effects of PTA and PNM, which was also confirmed by the receiver operating characteristic plot. The area under the curve (AUC) for the PTA × PNM interaction term was greater than the main effect of each variable (Figures 3A–C).

**Table 2:** Validation of the interaction effect using the Cox proportional hazard model.

Outcome	Model	Factor	B	HR	Wald	*p*-Value
LRFS	Original model	PNM	−0.203	0.816	7.383	**0** **.** **004**
	Interaction term added	PNM	−0.091	0.913	1.277	0.239
		PNM×PTA	−0.186	0.830	8.187	**0** **.** **004**
MFS	Original model	PTA	0.021	1.021	3.995	**0** **.** **046**
	Interaction term added	PTA	0.003	1.003	0.057	0.811
		PNM×PTA	−0.102	0.903	5.785	**0** **.** **016**
Overall survival	Original model	PTA	0.025	1.226	4.420	**0** **.** **036**
		Enneking staging	1.201	3.323	3.427	0.064
	Interaction term added	PTA	0.007	1.007	0.187	0.665
		Enneking staging	1.148	3.152	3.182	0.074
		PNM×PTA	−0.122	0.885	4.954	**0** **.** **026**

The multivariate analysis was repeated with the interaction term added to the Cox proportional hazard model. Significant factors are in bold. LRFS, local recurrence-free survival; MFS, metastasis-free survival; PTA, percentage of tumor cross-sectional area; PNM, peri-neurovascular margin; HR, hazard ratio.

## 4 Discussion

While a marginal margin is generally considered inadequate for *en bloc* resection of high-grade OS, the issue of “how close is too close” remains controversial (6). Conventionally, a >1–2 cm healthy cuff has been defined as “adequate” by Enneking et al. (8) and has been advocated by many reports (10, 11). A study of 1,126 subjects spanning 27 years of data also found that an inadequate margin resulted in a 23.5% 5-year LR rate vs. only 3.6% for an adequate, wide margin (12). However, this view has been radically challenged by other studies showing no significant difference in LR between margins greater than or less than 1 mm (2, 3), 5 mm (14), or with a super-close but pathologically negative margin (2, 14). This confusion may be further exacerbated by studies showing that a closer margin has no significant impact on patient survival in spite of higher local recurrence rates (27–29). A possible explanation for this discrepancy may be the different criteria used to define a “close margin,” which probably reflect the heterogeneous inclusion criteria in these reports. Considering the possible non-linear nature of tumor biology, in our study, we included those with an “inadequate wide margin (<1.5 cm)” without dichotomizing this variable using any *a priori* standard. This was particularly advantageous when using ML algorithms, which recursively compute all possibilities to optimize the final model and take interactions between factors into account (30). Interestingly, our results are in accordance with, at least in part, recent studies showing no effect of a pure margin on patient survival. However, the interaction between tumor size and margins robustly correlates with all the oncological indices, including LRFS, MFS, and overall survival, which has not been examined in previous reports. We posit that, when the tumor cross-sectional area is less than 31.6% of the limb, a wider PNM results in a better prognosis due to the curative effect of *en bloc* resection on the localized malignancies. In contrast, the same effect was not found when the tumor was bigger, probably due to the greater invasiveness of the tumor, which is not treatable by local intervention alone. Surprisingly, the interaction effect was found to be more significant compared with the main effect of each factor individually for LR, metastasis, and overall survival. To better visualize this interaction effect, the relationship between PNM, tumor size, and the Beta coefficient in the Cox proportional hazards model was plotted, as shown in Figure 3D.

Previous studies have quantified surgical margins through imaging (22), macroscopy (11, 22), and microscopy with (7, 14) or without (21) ink. In this study, high-resolution MRI was used to specifically measure peri-neurovascular margins, considering that PNM is the main cause of margin inadequacy in high-grade OS (7, 21–23) and is found to be more closely associated with oncological outcomes compared with soft tissue margins (22). MRI allows for an accurate examination of the relationship between the tumor and neurovascular bundle (31, 32) and is desirable for preoperative planning (33) compared with a pathology examination of the tissue after formalin fixation (22). Furthermore, quantification of the PNM based on a pathological biopsy could also potentially be biased if a surgeon performs an intensive dissection of the PNM in cases with a narrow peri-neurovascular fat layer but otherwise performs a relatively simple dissection in cases with an easily identifiable peri-neurovascular fat layer.

This study has several limitations. Response to neoadjuvant chemotherapy (tumor necrosis rate) and vessel sheath histological assessment data were not consistently available for all the patients in our cohort due to the retrospective nature of this study. In addition, since tumor necrosis rate is a postoperative parameter, it may have limited value in models aimed at guiding preoperative surgical planning. Additionally, the possibility of an apparent interaction effect arising from overfitting (false positive results) in the ML model was addressed using the cross-validation method in model generation and feature selection, and a relatively adequate sample size [>10–20 times the number of attributes (24, 34)]. A conventional Cox proportional hazards model was also used to validate our findings. Finally, although the decision tree model identified specific threshold values for PNM and PTA, these cutoff values were derived from a relatively small, single-center dataset with a limited sample size and therefore should be interpreted with caution. Rather, our findings challenge the traditional linear understanding of the risk related to margins by highlighting the prognostic value of margin width, depending on tumor burden, and considering tumor heterogeneity in the preplanning of surgical margins for high-grade extremity OS.

Our findings should not be interpreted as suggesting that pursuing high-quality surgical margins is negligible in larger tumors. Rather, the relatively poor outcome of peri-neurovascular margins in this subgroup may reflect the more aggressive biological behavior and higher systemic metastatic potential associated with larger tumor burdens. More aggressive multimodal systemic therapy in combination with local surgical control, rather than local control alone, is urgently needed. Further investigation with larger sample sizes and more advanced ML techniques is needed to explore tumor heterogeneity and precise pre-surgical planning in LSS for high-grade OS.

## Data Availability

The raw data supporting the conclusions of this article will be made available by the authors, without undue reservation.
