# Supplementary material for: A decision tree model suggests a strong interaction effect between tumor size and a close surgical margin on the prognosis of limb salvage surgery in high-grade osteosarcoma
Source: Front Surg. 2026 Apr 24;13:1801218. doi: 10.3389/fsurg.2026.1801218 (PMC13153076; doi:10.3389/fsurg.2026.1801218)
Supplement: Supplementary file 1 [file Supplementaryfile1.docx]

**Supplementary Methods 1：**

**Method for generating machine learning models with cross-validation(CV)**

For brief comparison of various machine learning (ML) model, namely, Naïve Bayes, Decision Tree (DT), Support Vector Machine (SVM) and Tree Bagger Algorithms, machine learning app in Matlab was used with all attributes as dependent variables and recurrence/ non-recurrence as dependent variable. No feature selection procedures were carried out at this stage. 5-fold cross validation method was used to prevent potential overtraining of model. Briefly, all samples were allocated into training set(4-fold) and a test set(1-fold), with the former fed into the model and the latter applied to the model as a test. This process was repeated for each one tenths of the samples in turn and the classification accuracy was averaged across all repeats. Because it is unknown whether the model and the according prediction accuracy were similar at different post-operative time, we fit the model with recurrence/ non-recurrence data at 12, 18, 24, 30, 40 months post-operatively. The sample size used for a given timepoint depends on the number of patients that were not censored until that time. For reference purpose, we also fit a cox proportional hazard regression model with DVT as the only variable. We then computed and thresholded the LR-free survival for each subject to obtain a predicted binary LR outcome. This predicted outcome was compared with true class in order to obtain the overall prediction accuracy.

**Selection of machine learning algorithms**

For brief comparison of various machine learning (ML) model, namely, Naïve Bayes, Decision Tree (DT), Support Vector Machine (SVM) and Tree Bagger Algorithms, machine learning app in Matlab was used with all attributes as dependent variables and recurrence/ non-recurrence as dependent variable. No feature selection procedures were carried out at this stage. 8-fold cross validation method was used to prevent potential overtraining of model. Briefly, all samples were allocated into training set(9-fold) and a test set(1-fold), with the former fed into the model and the latter applied to the model as a test. This process was repeated for each one tenths of the samples in turn and the classification accuracy was averaged across all repeats. Because it is unknown whether the model and the according prediction accuracy were similar at different post-operative time, we fit the model with recurrence/ non-recurrence data at 12, 18, 24, 30, 40 months post-operatively. The sample size used for a given timepoint depends on the number of patients that were not censored until that time. For reference purpose, we also fit a cox proportional hazard regression model with DVT as the only variable. We then computed and thresholded the LR-free survival for each subject to obtain a predicted binary LR outcome. This predicted outcome was compared with true class in order to obtain the overall prediction accuracy.

Our result indicated a roughly similar prediction accuracy at different timepoint among various ML algorithms **(Supplementary Table 1, Supplementary Fig1)**, however, the performance of cox proportional hazard model was generally inferior than ML algorithms, especially at 30 and 40 months after operation. Due the consideration that DT had a relative good prediction accuracy as well as being simplistic and easily interpretable, and that the superiority of DT model over conventional cox regression model began to be prominent at 30-month post-surgery. We subsequently look at DT model (30-months after surgery) with feature selection (pruning) based on the 8-fold cross validation method (wrapper algorithm) to fit our model.

DT model is one of the earliest technique and widely recognized as a very powerful ML technique[[1-4](#_ENREF_1)]. This model recursively examines each variable and corresponding threshold to find a variable-threshold pair that best classify the population into high- or low- risk LR subgroups using a tree-structured classification scheme. Breiman’s CART method[[5](#_ENREF_5), [6](#_ENREF_6)] was adopted in our study with Gini's diversity index as our splitting criterion. To remove the redundant variables (nodes) in the model, the minimal cross validation error was used as the pruning criterion to construct the final prognostic model.

In result, the unpruned DT model suggested DVT and PTA as the only parameters associated with poor prognosis of local recurrence **(Supplementary Fig2.A).** After pruning, DVT (whether≥2.4mm) and PTA (whether<31%) remain in the final DT model **(Supplementary Fig2. B, C).**

**Multicollinearity Problem**

To ensure the stability of the Cox proportional hazards regression model, multicollinearity among candidate variables was assessed by examining pairwise correlation coefficients and variance inflation factors (VIF). Variables with correlation coefficients greater than 0.7 or VIF values greater than 5 were not simultaneously included in the final regression model. Among tumor morphology–related parameters (tumor volume, axial length, cross-sectional area, and percentage of tumor cross-sectional area [PTA]), only one representative variable was retained when strong correlations were present. Multicollinearity assessment was primarily performed for the Cox regression model rather than the machine learning algorithms, as regression-based models are more sensitive to collinearity among predictors. In contrast, the Decision Tree (DT) algorithm is relatively robust to multicollinearity because variables are selected sequentially during node splitting, and highly correlated predictors generally do not destabilize the model[7]. In addition, cross-validation–based feature selection was applied in the machine learning workflow to further reduce redundancy among candidate variables. The selection of PTA by DT as the representative feature is also logically straightforward, since it is normalized to the limb cross-sectional area and therefore less affected by variations in limb size or skeletal dimensions, providing a relatively standardized measure of tumor burden.

1. Nunn ME, Fan J, Su X, Levine RA, Lee HJ, McGuire MK. Development of prognostic indicators using classification and regression trees for survival. Periodontol 2000 2012;58(1):134-42.

2. Schilling C, Mortimer D, Dalziel K, Heeley E, Chalmers J, Clarke P. Using Classification and Regression Trees (CART) to Identify Prescribing Thresholds for Cardiovascular Disease. Pharmacoeconomics 2015.

3. Taghipour Zahir S, Binesh F, Mirouliaei M, Khajeh E, Noshad S. Malignancy risk assessment in patients with thyroid nodules using classification and regression trees. J Thyroid Res 2013;2013:983953.

4. Temkin NR, Holubkov R, Machamer JE, Winn HR, Dikmen SS. Classification and regression trees (CART) for prediction of function at 1 year following head trauma. J Neurosurg 1995;82(5):764-71.

5. Marshall RJ. The use of classification and regression trees in clinical epidemiology. J Clin Epidemiol 2001;54(6):603-9.

6. Speybroeck N. Classification and regression trees. Int J Public Health 2012;57(1):243-6.

7. Kononenko I. Machine learning for medical diagnosis: history, state of the art and perspective. Artificial Intelligence in Medicine 2001; 23 (1): 89-109.
