# Supplementary material for: A decision tree model suggests a strong interaction effect between tumor size and a close surgical margin on the prognosis of limb salvage surgery in high-grade osteosarcoma
Source: Front Surg. 2026 Apr 24;13:1801218. doi: 10.3389/fsurg.2026.1801218 (PMC13153076; doi:10.3389/fsurg.2026.1801218)
Supplement: Supplementary file 2 [file Table1.doc]

**Supplementary Tables and Figure Captions：**

**Table S1** Comparison of ML algorithms and cox proportional hazard model

| Prediction model | Prediction accuracy | | | | |
| --- | --- | --- | --- | --- | --- |
|  | 12 mo | 18mo | 24mo | 30mo | 40mo |
| Naïve Bayes Classifier | 0.835 | 0.776 | 0.746 | 0.698 | 0.729 |
| Naïve Bayes | 0.835 | 0.761 | 0.746 | 0.679 | 0.688 |
| Decision Tree | 0.847 | 0.836 | 0.762 | 0.660 | 0.771 |
| SVM | 0.871 | 0.776 | 0.746 | 0.660 | 0.625 |
| Tree Bagger | 0.812 | 0.806 | 0.667 | 0.623 | 0.688 |
| Cox proportional hazard model | 0.847 | 0.746 | 0.667 | 0.547 | 0.520 |

**Supplementary Fig.1 Margin status and oncological outcomes in limb salvage surgery(LSS) with Close surgical margin(CSM)** In the 88 cases with en-bloc(wide) resection of tumor, peri-neurovascular margin(PNM) was less than 6 mm in the majority (51 of 88) **(a)**. This cohort had a 5 year local recurrence (LR) free survival rate of 0.65(0.07) , metastasis free survival rate of 0.56(0.06) **(b)**, and overall survival probability of 0.64(0.07) **(c).** Probability was estimated by Kaplan-Meier analysis and expressed as mean (standard error).

**Supplementary Fig 2 Comparison of machine learning(ML) algorithms and cox proportional hazard model.** A roughly similar prediction accuracy could be achieved at different timepoint among various ML algorithms. However, the performance of cox proportional hazard model was inferior, especially 30 and 40 months after operation, than ML algorithms.

**Supplementary Fig 3 Constructing the final DT model using cross-validation.** The unpruned DT model was fitted to predict the risk of local recurrence of osteosarcoma at 30-months after surgery**(a)**. To avoid the overtraining of the model, feature selection (pruning) based on the 5-fold cross validation method suggested a number of 3 terminal nodes have a good model generalizability, as it minimize the cross validation error in addition to the resubstitution error**(b).** The removed branches were shown as dotted lines in **a** and the final pruned DT model was shown in **c**.
