# Supplementary figures and images for: A decision tree model suggests a strong interaction effect between tumor size and a close surgical margin on the prognosis of limb salvage surgery in high-grade osteosarcoma
Source: Front Surg. 2026 Apr 24;13:1801218. doi: 10.3389/fsurg.2026.1801218 (PMC13153076; doi:10.3389/fsurg.2026.1801218)

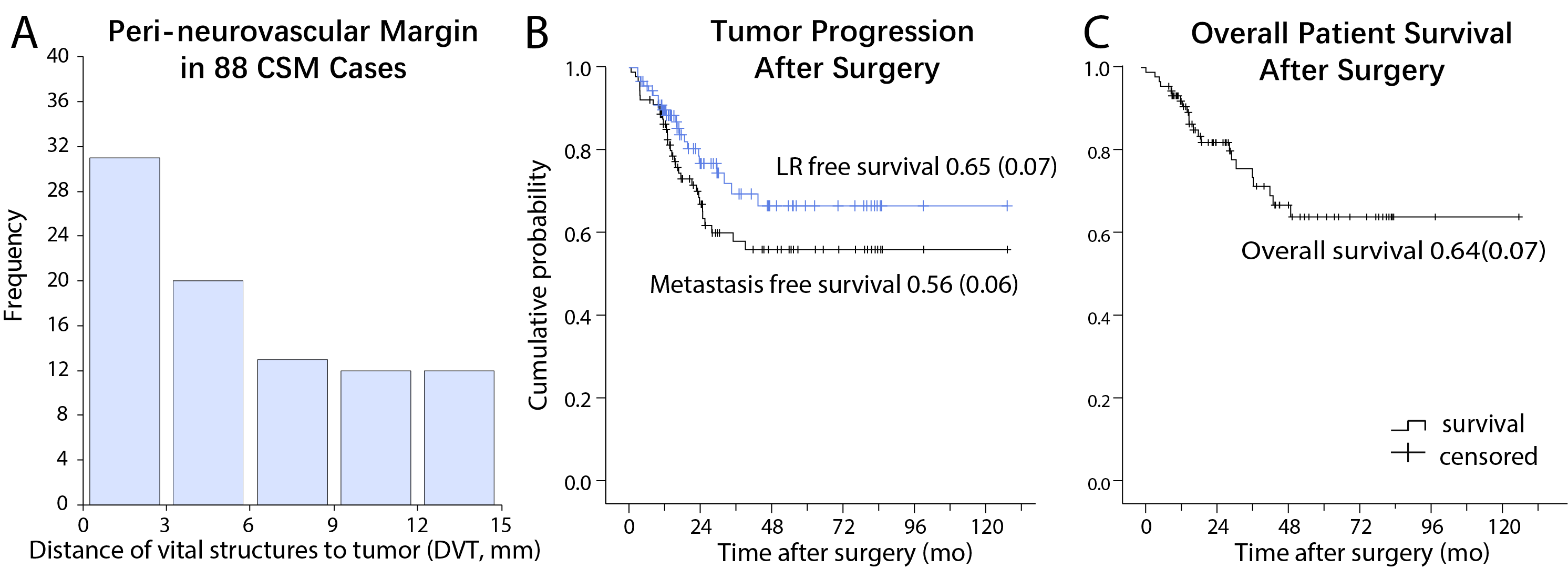

Supplement: Supplementary file 3 [file Image1.tif]

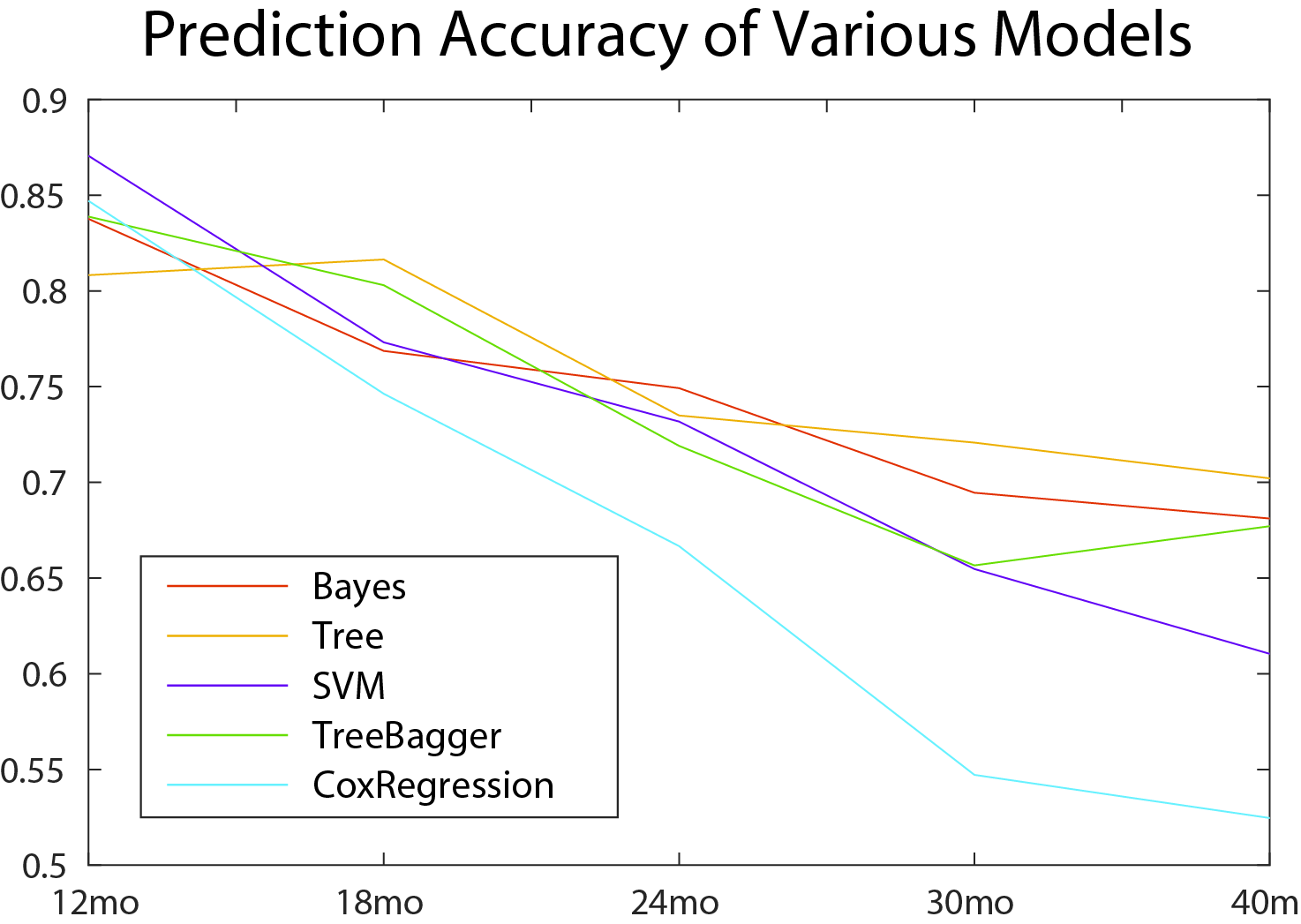

Supplement: Supplementary file 4 [file Image2.tif]

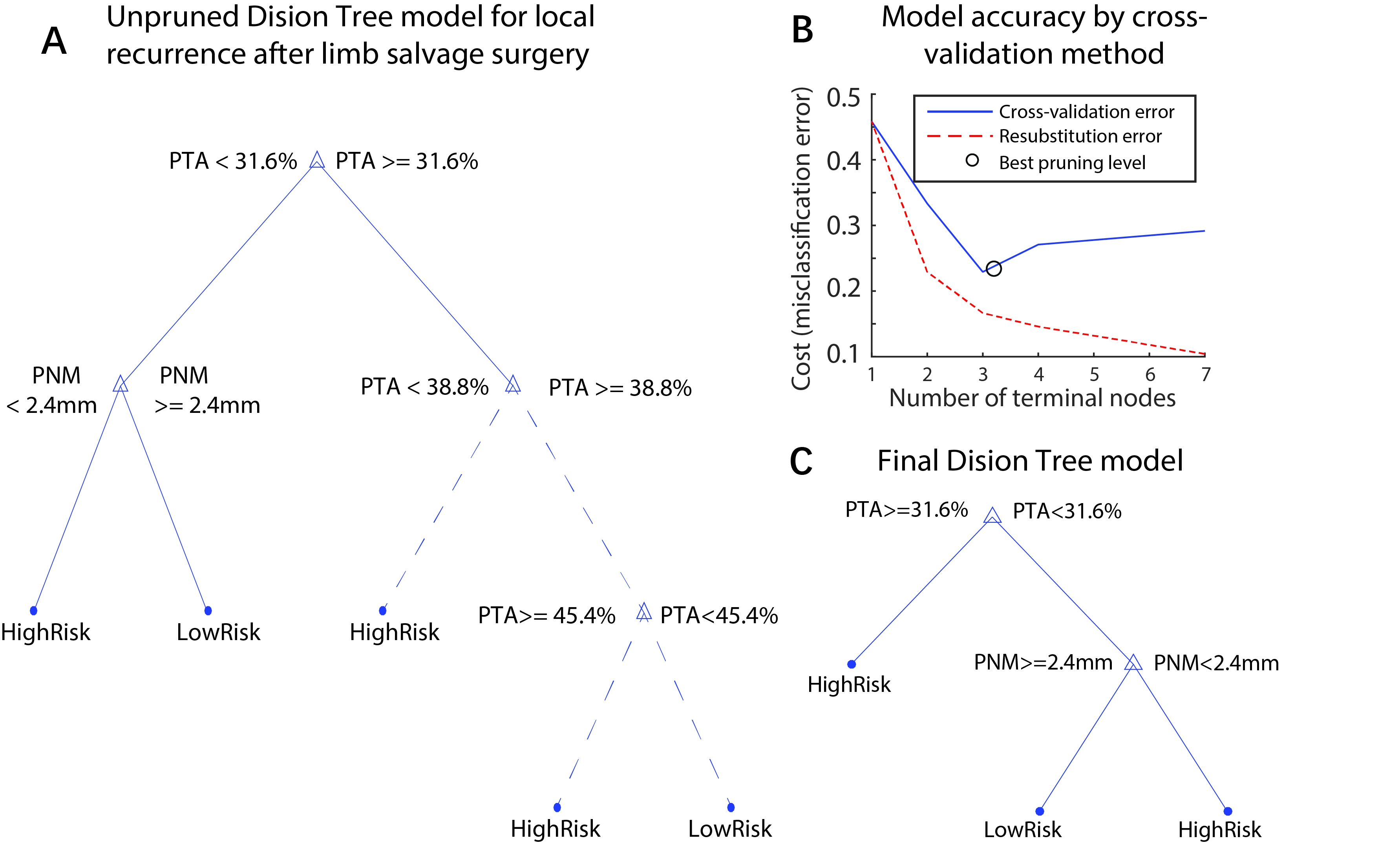

Supplement: Supplementary file 5 [file Image3.tif]
